# Electrochemical and Biological Performance of Biodegradable Polymer Coatings on Ti6Al7Nb Alloy

**DOI:** 10.3390/ma13071758

**Published:** 2020-04-09

**Authors:** Wojciech Kajzer, Janusz Szewczenko, Anita Kajzer, Marcin Basiaga, Marcin Kaczmarek, Magdalena Antonowicz, Joanna Jaworska, Katarzyna Jelonek, Arkadiusz Orchel, Katarzyna Nowińska, Janusz Kasperczyk

**Affiliations:** 1 Department of Biomaterials and Medical Devices Engineering, Faculty of Biomedical Engineering, Silesian University of Technology, 41-800 Zabrze, Poland; 2 Centre of Polymer and Carbon Materials of the Polish Academy of Sciences, 41-819 Zabrze, Poland; 3 Department of Biopharmacy, Faculty of Pharmaceutical Sciences in Sosnowiec, Medical University of Silesia, 40-055 Katowice, Poland; 4 Faculty of Mining and Geology, Department of Applied Geology, Silesian University of Technology, 44-100 Gliwice, Poland

**Keywords:** Ti6Al7Nb, drug-eluting polymer coatings, wettability, corrosion resistance, metallic ion release, cytotoxicity

## Abstract

The inhibition of the corrosion of metal implants is still a challenge. This study aimed to increase the corrosion resistance of Ti6Al7Nb alloy implants through surface modification, including grinding, sandblasting, and anodic oxidation followed by the deposition of a polymer coating. The aim of the work was to determine the influence of biodegradable polymer coatings on the physico-chemical properties of a Ti6Al7Nb alloy used for short-term implants. Biodegradable coatings prepared from poly(glycolide-caprolactone) (P(GCap)), poly(glycolide ε-caprolactone-lactide) (P(GCapL)), and poly(lactide-glycolide) (PLGA) were applied in the studies. The dip-coating method with three cycles of dipping was applied. Corrosion resistance was assessed on the basis of potentiodynamic studies. The studies were carried out on samples after 30, 60, and 90 days of exposure to Ringer’s solution. Surface topography, wettability, and cytotoxicity studies were also carried out. The degradation process of the base material was evaluated on the basis of the mass density of the metal ions released to the solution. The results indicated the influence of the coating type on corrosion resistance. In addition, a beneficial effect of the polymer coating on the reduction of the density of the released metal ions was found, as compared to the samples without polymer coatings. The obtained results provide basic knowledge for the development of polymer coatings enriched with an active substance. The presence of ciprofloxacin in the coating did not reduce the corrosion resistance of the metal substrate. Moreover, the cytotoxicity test using the extract dilution method demonstrated that the implants’ coatings are promising for further in vitro and in vivo studies.

## 1. Introduction

In the last few decades, dynamic research on a variety of polymer biomaterials has been observed. This was mainly caused by the satisfactory biocompatibility of biopolymers and their dedicated functionality. The group of synthetic bioresorbable polymers—for example, polylactide (PLA), poly(ε-caprolactone (PCL), and poly(glycolide) (PGA)—is particularly interesting for researchers [1,2,3,4]. The extensive use of biodegradable polymers in medical applications is mainly associated with the possibility of tailoring their physicochemical and mechanical properties. Controlled polymer degradation allows for their use as carriers of medicinal substances, ensuring drug release with regulated dynamics to obtain the expected therapeutic effects [5]. The mentioned group of biopolymers is characterized by an appropriate biocompatibility [4], and the achieved degradation products—mainly lactic, glycolic, and hydroxyhexanoic acids—are found to be neutral to the human organism and are metabolized in accordance the Krebs cycle [6]. The basic limitation of the use of biodegradable polymers relates to the mechanical properties that change over time. This feature may be disadvantageous in some applications, e.g., orthopedic implants. Treatment disorders can be observed as the implant weakens due to degradation processes. This can lead to repeated injuries. The consequence of this is the need to perform a revision surgery combined with the removal of an incompletely degraded and mechanically weakened implant and followed by its replacement with a new one. The revision surgery due to the progressive degradation of the polymer material may be associated with serious difficulties that lead to a much greater traumatization of the surrounding tissue with reference to the original implantation procedure. Therefore, despite the continuous development of material science and engineering, which is fundamental for the development of a wide range of biomaterials, metal materials are still the primary group. Their biological performance and universality are the result of long-term clinical outcomes and relatively low manufacturing and use costs. Due to their relatively high and durable mechanical properties, metal materials have a good long-time performance. However, metal biomaterials are characterized by a relatively low biocompatibility. Combined with the environment of tissues, body fluids, and mechanical factors, metals undergo corrosive destruction [6,7,8]. Compared to other metal biomaterials, titanium alloys show good mechanical properties that are responsible for the transfer of static and dynamic loads. One of the most popular Ti-based alloys is the Ti6Al4V alloy. However, based on recent research, it is needed to be mentioned that, despite being the first choice material in orthopedic applications, the alloy often causes allergic reactions due to the presence of vanadium, aluminum, and titanium [9,10,11]. Therefore, the Ti6Al7Nb alloy was developed for improved biocompatibility. Due to the replacement of toxic vanadium with neutral niobium, an increase in corrosion resistance and biocompatibility was obtained. However, the toxic/allergic interaction of aluminum and titanium ions has remained an unresolved problem. Therefore, the basic direction of current research on improving the biocompatibility of metal biomaterials, including titanium alloys, is to modify their surface layers [10,12]. Anodic oxidation is a commonly used method of the surface modification of titanium alloys [13]. Interesting results have also been obtained by applying SiO_2_ or TiO_2_ coatings produced by the sol–gel and atomic layer deposition (ALD) methods [1,9,14,15,16,17,18]. However, despite the broad number of studies in this area, the problem of the harmful influence of the degradation products of metal biomaterials have yet to be fully determined.

Hence, the use of biodegradable polymer coatings is a promising method to modify the surface of metal implants, ensuring conditions for the correct course of treatment. The applied metal is expected to provide an appropriate mechanical stability of the implant throughout the whole treatment period. In contrast, the polymer coating should be considered as a barrier to the degradation products of the metal biomaterial. In addition, biodegradable polymer coatings containing drugs, e.g., antibiotics like ciprofloxacin, can support the healing process through the local and controlled release of active substances. This applies, in particular, to short-term implants for which no osseointegration occurs and which are removed from the body after the treatment process. The local delivery of medicinal substances contributes to their more effective impact, reduces the risk of local infection, and, at the same time, limits the number of medicinal substances used compared to traditional pharmacotherapy [5,18,19]. 

Furthermore, the polymer coating that forms the intermediate layer between the metal substrate and the body’s environment limits implant osseointegration, minimizing tissue traumatization during implant removal after the finished treatment.

Aliphatic polyesters—mainly including PGA and PLA, as well as random block copolymers poly(lactide-co-glycolide) (PLGA) and poly(ε-caprolactone) (ε-PCL)—were among the few synthetic biodegradable polymers approved for human clinical use due to their biocompatibility and biodegradation. They were first applied for biodegradable sutures and later for temporary prostheses, 3D porous scaffolds applied in tissue engineering, regenerative medicine, bionanotechnology, gene therapy, the controlled release of drugs, and implantable orthopedic devices. The degradation of aliphatic polyesters occurs by the hydrolysis of labile ester bonds. The degradation products (carbon dioxide and water) are metabolized by the organism [18,20,21,22,23,24,25,26]. The important factors that influence the rate of the degradation are molecular weight, the copolymer composition of the polymer chain, shape, glass transition temperature, and crystallinity. PCL is a semicrystalline polyester that has flexible mechanical properties with a quite long degradation rate in the range of two-to-three years for pure polycaprolactone products. Therefore, polycaprolactone is frequently copolymerized or blended with other polymers to increase its degradation rate or to modify its physicochemical properties, e.g., the copolymers of ε-caprolactone and glycolide are characterized by lower stiffness than products obtained from polyglycolide [22].

Previous studies on the impact of polymer coatings on the corrosion resistance of a metal substrate, mainly non-biodegradable coatings, have been performed [23,27,28]. However, there have been a limited number of reports on the impact of biodegradable coatings on the corrosion resistance of metal substrates and their cytotoxicities [18,19,29,30,31].

Therefore, the main purpose of the work was to determine the impact of biodegradable polymer coatings and the method of their application (the number of dips in the dip-coating method) on the physicochemical properties and cytotoxicity of the developed systems intended for short-term therapy—presented in the study over three of months of exposure to Ringer’s solution. Poly (glycolide-ɛ-caprolactone) (P(GCap)), poly (glycolide-ɛ-caprolactone-L, L-lactide) (P(GCapL)), and PLGA were used as coatings. All applied polymer layers contained an antibacterial agent—ciprofloxacin (CFX). CFX, as a second generation fluoroquinolone, is used to treat various kinds of infections including urinary tract, bone, and joint infections [32]. It is believed that its local release may prevent or overcome the possible infections of an implantation procedure. In our previous study, the ciprofloxacin release profiles from biodegradable coatings were characterized, and the antibacterial activity of the release drug was confirmed [33]. Moreover, in the current study, the barrier properties of the developed polymeric layers, analyzed on the basis of the density of the metal ions released to the medium, were confirmed. It should be emphasized that the ability to limit undesirable metal ions was maintained even after three months of exposure to a medium. Based on microscopic observations, the degradation process of polymer coatings was assessed, as was their wettability and surface topography as a function of time of exposure to a corrosive solution. 

## 2. Materials and Methods 

A Ti6Al7Nb alloy was the material used in the studies. The alloy fulfilled the requirements of the ISO 5832-11 standard [34] with reference to chemical composition, microstructure, and mechanical properties. The samples in the form of discs were taken from rods with diameters of 24 and 6 mm and a length of 60 mm. The study used each type of samples for which the subsequent surface modification treatments were carried out: grinding, sandblasting, and anodic oxidation. For grinding, abrasive papers of the 120, 300, and 600 grades were used. The sandblasting process was carried out using glass balls with diameters ranging from 70 to 110 μm. The applied time was equal to t = 2 minutes. For the final surface treatment—anodization—an electrolyte containing phosphorous and sulphuric acids was applied. The applied parameters of the process were voltage equal to V = 97 V and time equal to t = 2 min. 

Different kinds of polymers were used to prepare the matrix with ciprofloxacin. The P(GCap) (10/90), P(GCapL) (10/12/78), and PLGA (84/16) copolymers were synthesized in bulk by the ring opening polymerization (ROP) of ɛ-caprolactone, glycolide, L-lactide, and D,L-lactide at 120 °C for 96 h (P(GCapL)) and at 120 °C for 72 h (PLGA and P(GCap)) in an argon atmosphere using zirconium (IV) acetylacetonate (Zr(acac)_4_) as a non-toxic initiator with an initiator/monomers molar ratio of 1/1200 [35]. The obtained materials for purification were dissolved in chloroform and precipitated in cold methanol. The purified materials were dried at room temperature to a constant weight (in a vacuum). The polymer coatings were prepared according to the dip-coating method (dip-coater, MTI Corporation) with 3 cycles of dipping in a 1% polymer solution in CH_2_Cl_2_ (w/w) containing 20% of ciprofloxacin (w/w). Applied immersion time was 30 s, and the delay between dips was 15 min.

### 2.1. Surface Roughness

A topological study of the metal substrate and the polymer coatings were carried out with a Sensofar Sneox optical profilometer. A combination of two scanning techniques was applied: confocal and focal differentiation for each measured frame (Confocal Fusion). Light with a wavelength of 530 nm and a 20× magnification lens were used. The tests were carried out for samples in the initial state and after 3 months of exposure to Ringer’s solution.

### 2.2. Microscopic Observations

The morphology of the samples before and after the exposure to Ringer’s solution was analyzed with a Zeiss Stereo Discovery V8 stereoscopic microscope (Zeiss, Oberkochen Germany) with a MC5s digital camera. 

### 2.3. Wettability Test

The wettability of the polymer coatings was determined based on the measurement of the contact angle with the use of a Surftens Universal goniometer (OEG, Frankfurt (Oder), Germany) equipped with the Surftens 4.3 software. The volume of the dosing drop of the demineralized water was 1 µm^3^; the measurement was initiated 10 s after the drop was deposited. The measurements were carried out at room temperature (T = 23 ± 1 °C) over 60 s with a sampling rate of 1 Hz. The tests were carried out for samples in the initial state and after different exposure times to Ringer’s solution.

### 2.4. Potentiodynamic Test

The resistance to pitting corrosion was tested by the potentiodynamic method, meeting the requirements of PN-EN ISO 10993-15 standard [36], with the use of a Voltalab PGP201potentiostat (Radiometer, Villeurbanne Cedex, France). The reference electrode was that of the Ag/AgCl 3M KCl, while the auxiliary one was a platinum rod. 

Corrosion tests were started by determining the E_ocp_ open circuit potential in currentless conditions, and then polarization curves were recorded. Polarization curves were recorded from the value of the initial potential E_start_ = E_ocp_ – 100 mV. The scan rate was equal to 3 mV/s. After reaching the potential of E = 4 V, the polarization direction was changed. On the basis of the obtained curves using the Stern method, the corrosion potential E_corr_ and the value of polarization resistance R_p_ were determined.

The tests were carried out in a Ringer’s solution with the following chemical composition—NaCl: 8.6 g/dm^3^; KCl: 0.3 g/dm^3^; and CaCl_2_ 2H_2_O: 0.33 g/dm^3^—at the temperature of T = 37 ± 1 °C and pH = 6.9 ± 0.2. The tests were carried out for both the non-coated samples (substrate) and the samples with polymer coatings in the initial state and after 1, 2, and 3 months of exposure to Ringer’s solution.

### 2.5. Ion Release Test

Metal ion concentration in Ringer’s solution after 1, 2, and 3 months of exposure was measured with a JY 2000 spectrometer by Jobin-Yvon Horiba (Kyoto, Japan) using inductively coupled plasma-atomic emission spectrometry (ICP-AES). The tests were carried out for both the non-coated and the coated samples. The number of metallic ions released from the surface of the samples to the solution were converted into surface mass density μg/cm^2^.

### 2.6. Biological Test

Sample extract preparation: The cytotoxicity test was performed according to the ISO 10993-5 standard [37] using the extract dilution method. The extracts were prepared throught the incubation of implants with drug-free polymer coatings (GCap, GCapL, and PLGA) or implants with polymer coatings containing a ciprofloxacin (GCap and Cfx, GCapL and Cfx, and PLGA and Cfx) culture medium (minimum essential medium Eagle; MEM, Sigma-Aldrich). The implants were incubated at 37 °C for 24 and 72 h. Aliquots of each preparation were collected and prepared in 3 concentrations: 100%, 33.3%, and 20.0%. The extracts were diluted with MEM. 

Cell culture: Human chondrocytes (Lonza, Switzerland) were cultured in a medium containing minimum essential medium Eagle (MEM, Sigma-Aldrich), 10% fetal bovine serum (Thermo Fisher Scientific), 100 U/ml penicillin, 100 μg/mL streptomycin (Sigma-Aldrich), and 10 mM HEPES (4-(2-Hydroxyethyl)piperazine-1-ethanesulfonic acid) (Sigma-Aldrich). The cells were grown in 25 cm^2^ tissue culture flasks (Nunc) at 37 °C in a humidified atmosphere of 5% CO_2_/95% air. For the proliferation study, cells were seeded at an initial density of 10^3^ cells/well in clear 96-well plates and cultured for 24 h. Then, the culture medium was removed and replaced with 0.2 mL of extract at concentrations of 100%, 33.3%, and 20.0%. Cell incubation then proceeded for 72 h. For the negative control, untreated cells were used. For the positive control group, the MEM with 5% of dimethyl sulfoxide (DMSO) was used.

Sulforhodamine B (SRB) assay: After incubation, the medium was removed, and the cells were fixed with 10% trichloroacetic acid. Then, the wells were washed with deionized water and stained with 0.4% sulforhodamine B (SRB, Sigma-Aldrich) dissolved in 1.0% acetic acid. Subsequently, plates were washed three times with 1% acetic acid, and the incorporated dye was dissolved in 200 μL of 10 mM Tris base solution. Absorbance was measured at 570 and 690 nm (reference wavelength) with an MRX Revelation plate reader (Dynex Technologies).

### 2.7. Statistical Analysis

The physical and electrochemical tests results are presented as means with standard deviation. In order to determine the significance of differences for *p* < 0.05, the obtained results used a one-way and two-way analysis of variance (ANOVA). All biological experiments were realized in triplicate. Statistically significant differences between groups were calculated using a one-way ANOVA followed by post-hoc Tukey test. To determine the homogeneity of variance, the Brown–Forsyth test was used. Statistical significance as before was declared at *p* < 0.05.

## 3. Results and Discussion

### 3.1. Surface Roughness

The determined parameters of the polymer coating roughness obtained after one dip not exposed to Ringer’s solution (0 month—NoE (no exposition)) regardless of the type of polymer— PLGA, P(GCap), or P(GCapL)—were slightly lower than the roughness of the metal substrate (Sa = 0.47 ± 0.02 m); see Table 1. Similar results were observed for poly(lactide-co-glycolide) with an equimolar amount of comonomeric units [38]. In addition, the increase in the number of dips (two and three dips) did not significantly change the registered value of the Sa parameter (*p* > 0.05) compared to the coatings obtained after one dip. After three months of exposure to Ringer’s solution, a small increase in surface roughness was observed for most coatings and their variants. The recorded change in the Sa parameter value for these samples, as in the case of a direct comparison of the impact of the number of dips on the Sa parameter of the samples at the initial state, was not statistically significant (*p* > 0.05).

### 3.2. Microscopic Observations Results

**Figure 1:**
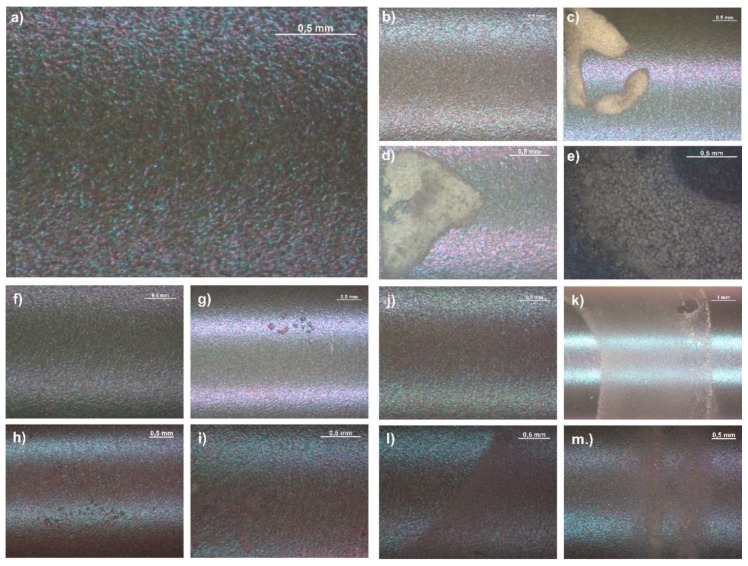
Surface of the: (**a**) sandblasted and oxidized Ti6Al7Nb; (**b**) P(GCap) polymer coating obtained after 1 dip—the sample that was not exposed to Ringer’s solution (NoE); (**c**) P(GCap) (1 dip) after 1 month of exposure to Ringer’s solution (1 m); (**d**) P(GCap) (1 dip) (2 m); (**e**) P(GCap) (1 dip) (3 m); (**f**) P(GCap-L) (2 dips)—NoE; (**g**) P(GCap-L) (2 dips) (1 m); (**h**) P(GCap-L) (2 dips) (2 m); (**i**) P(GCap-L) (2 dips) (3 m); (**j**) PLGA (3 dips)—NoE; (**k**) PLGA (3 dips) (1 m); (**l**) PLGA (3 dips) (2 m); and (**m**) PLGA (3 dips) (3 m).

On the surface of the Ti6Al7Nb alloy, there were evenly distributed traces characteristic for the anodic oxidation of the sandblasting process prior to oxidation (Figure 1a). The polymeric coatings with a drug applied on the metal substrate were characterized by translucency, homogeneity, and continuity over the whole surface of the samples, regardless of the type of polymer and its application parameters (Figure 1b,f,j). The applied coatings mapped the topography of the substrate. However, the local discoloration (transparency reduction) of the coating was observed as a result of exposure to Ringer’s solution. This effect might be associated with the appearance of oligomers, which tend to crystallize. This effect is well-known and has been described in the literature [39,40]. The area of the discoloration of the polymer coatings increased over time (Figure 1c–e,g–i,k–m).

### 3.3. Wettability Test

**Figure 2:**
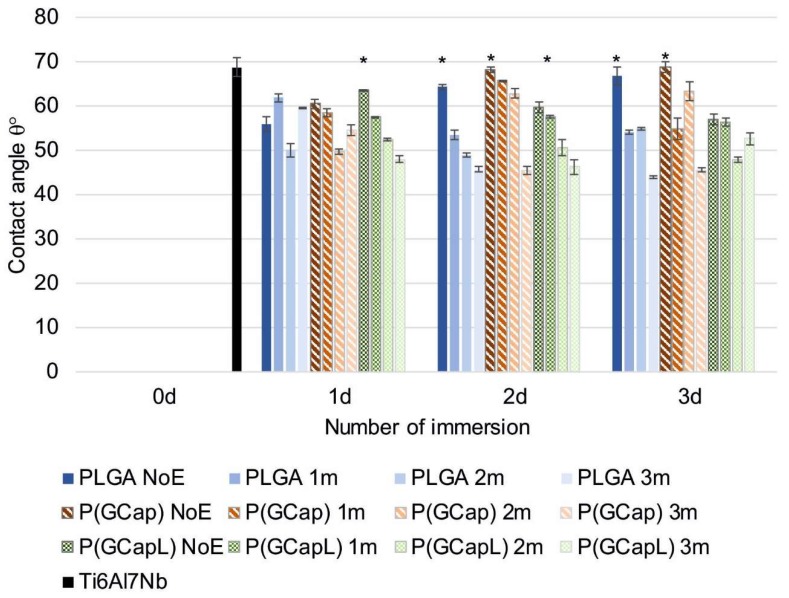
Wettability test results for samples after different numbers of dips and times of exposition to Ringer’s solution for the PLGA, P(GCap), and P(GCapL) coatings (* *p* < 0.05 versus the control group).

The results of measurements of the wetting angle of polymer coatings are shown in Figure 2. Wettability and hydrophilicity are important properties of biomaterials because they have a great influence on the cell’s adhesion, proliferation, and growth [41]. Regardless of the type of polymer coating and the method of its obtaining (number of dips), they were all hydrophilic. The applied coatings were characterized by a greater wettability than the Ti6Al7Nb substrate after the sandblasting and anodization processes, for which the contact angle value was 68.7 ± 2.1°. The exception was the poly(glycolide-ɛ-caprolactone) P(GCap) coatings obtained after two and three dips, for which the contact angles were comparable to the contact angles of the substrate. This effect might be associated with the presence of caprolactone segments (the content of ɛ-caprolactone units was 90%) due to the hydrophobicity of poly(ɛ-caprolactone) [42]. The analysis of the impact of the number of dips in the process of applying polymer coatings on the metal substrate showed its significance on the obtained contact angle values (*p* < 0.05). For PLGA and P(GCap) coatings obtained after two and three dips, a significant reduction in the wettability of the surface relative to the wettability of the coating obtained after one dip (*p* < 0.05) was observed. For the samples that were not exposed to Ringer’s solution, the angle values were 64.29° (PLGA two dips) and 66.8° (PLGA three dips) compared to the PLGA polymer coating obtained after a single dip, for which the angle value was 55.9°. The same relationship was observed for the P(GCap) coating. The contact angle values for two and three dips were 68.2° and 68.8°, respectively, compared to a polymer coating obtained by one dip, for which the contact angle was 60.41°. However, for the poly(glycolide-ɛ-caprolactone-L,L-lactide) P(GCapL) coatings, which contained 74 mol.% of L,L-lactide and were obtained by two and three dips, a significant (*p* < 0.05) increase in the hydrophilic properties of the surface was observed. The obtained contact angle values were equal to: two dips = 59.6° and three dips = 56.9°, with the reference to the coating after one dip (63.4°).

The surface wettability increased for most coatings as a result of one, two, and three months of exposure to Ringer’s solution, which may indicate swelling of the coatings. It can be also connected with the acidic products of the degradation of the polymer chain, which appeared on the surface of and within the material. The process of formation of acidic products during PLA degradation was described in detail by Li S. [41]. Only in the case of the PLGA and P(GCap) coatings obtained as a result of a single immersion (one dip) and P(GCapL) obtained as a result of triple immersions (three dips) was a similar wettability observed throughout the exposure period (*p* > 0.05). For the remaining analyzed variants, a significant influence of exposure time on the change in wetting angle was found (*p* < 0.05). For example, for the P(GCapL) coating obtained by one dip, the contact angle value decreased from 63.5° for the non-exposed surface to 47.7° after three months of exposure (*p* < 0.05). However, for the same polymer coating obtained with additional layer (two dips), the angle value changed from 59.6° to 46.2° (*p* < 0.05). For the P(GCap) coating after three months of exposure, a significant (*p* < 0.05) increase in wettability was also observe, from 68.2° for the non-exposed coating to 45.3° (two dips) and from 68.8° to 45.4° (three dips). However, for the PLGA polymer, the contact angle value for the unexposed coating obtained by one dip was 55.9° and increased insignificantly to 59.5° for the coating after three months of exposure (*p* > 0.05). For the same coating obtained by two and three dips, the tendency to change the angle was the same as for the -P(GCap) coating. A significant increase in the wettability of the surface was observed (*p* < 0.05), and angle values changed from 64.3° to 45.6° (two dips) and from 66.8° to 43.9° (three dips). As mentioned above, the increase in the wettability of the coating resulted from the degradation process of the aliphatic polyesters—the weak ester bonds present in the polymer chain were broken down, which generated the formation of short chains of oligomers at an advanced stage of degradation and, hence, an increase in the number of hydroxyl groups. 

### 3.4. Potentiodynamic Tests Results

**Figure 3:**
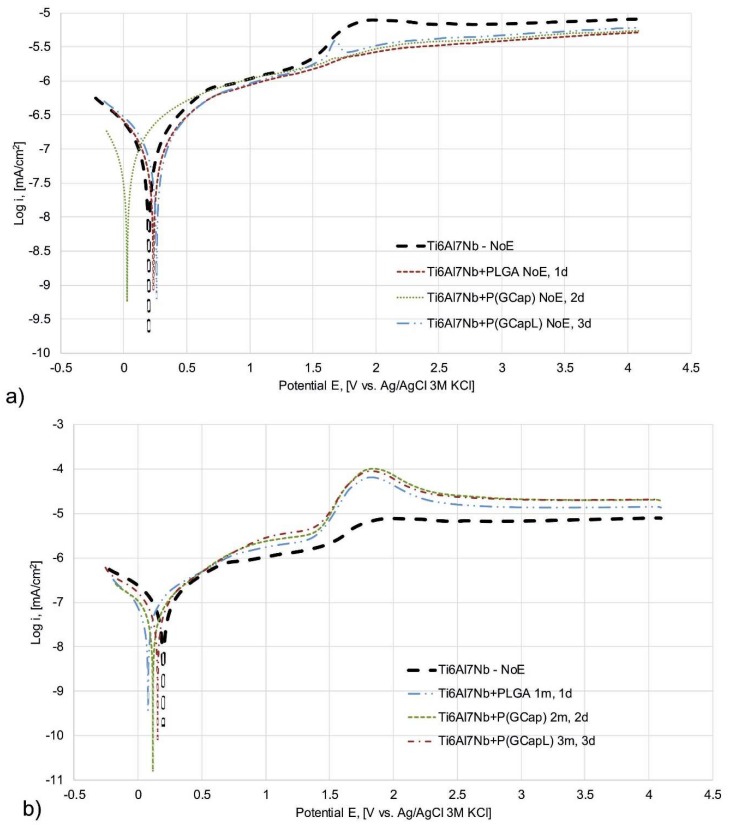
Sample polarization curves of Ti6Al7Nb in the initial state and the alloy with: (**a**) PLGA, P(GCap), and P(GCapL) coatings obtained as a result of different number of dips—1, 2, and 3, respectively (no exposition to Ringer’s solution—NoE); (**b**) Ti6Al7Nb—NoE, PLGA (1 dip; 1 m); P(GCap) (2 dips; 2 m) and P(GCapL) (3 dips; 3 m) polymer coatings.

The potentiodynamic results of the Ti6Al7Nb alloy with coatings deposited after one, two, and three times immersion in PLGA, P(GCap), and P(GCapL) at the initial state (NoE) and after prolonged exposure to Ringer’s solution are summarized in Table 2 and Figure 3.

Polarization curves were recorded for all analyzed samples. No breakdown potential was observed. This demonstrated the resistance to pitting corrosion. The lack of breakdown potential suggested a resistance to pitting corrosion. However, to prove the resistance, the study had to be supplemented by microscopic observations of the tested samples. The authors found no corrosion damage and found a good corrosion resistance of the anodized Ti6Al7Nb alloy [43]. For all tested variants with polymer coatings, registered potentiodynamic curves were characterized by a flat course indicating perfect passivation in the entire measuring range (+4000 mV). However, for the anodized Ti6Al7Nb samples without the polymer coating, a slight increase in current density was observed in the potential range from 1.5 to 2.0 V (Figure 3a) due to the remodeling of the oxide layer on the Ti alloy surface [44,45,46]. 

The analysis of the course of the sample polarization curves clearly indicated that the application of polymer coatings to the metal substrate, regardless of their type and application parameters, reduced the current density in the entire measurement range compared to the metal substrate (Figure 3a). With the increase of the exposure time of samples with the polymer coatings, regardless of their type and application parameters, an increase in current density over the entire measurement range was observed compared to the non-exposed samples (Figure 3b). Moreover, in the 1.5 to 2 V potential range for the samples after one, two, and three months of exposure, a distinct increase in the current density was observed [43,44]. This remodeling indicated the progressive degradation of the polymer coatings and, thus, the increasing contact of the metal substrate with the surrounding corrosive environment. Moreover, the effect of long-term exposure to Ringer’s solution was insignificant (*p* > 0.05) for the value of the recorded corrosion potentials both for the metal substrate and coated samples, regardless of the type of the polymer and application parameters. Moreover, no explicit influence of exposure time, coating type, and application parameters on the value of polarization resistance (R_p_) was found (*p* > 0.05). 

### 3.5. Ion Release Test Results

**Figure 4:**
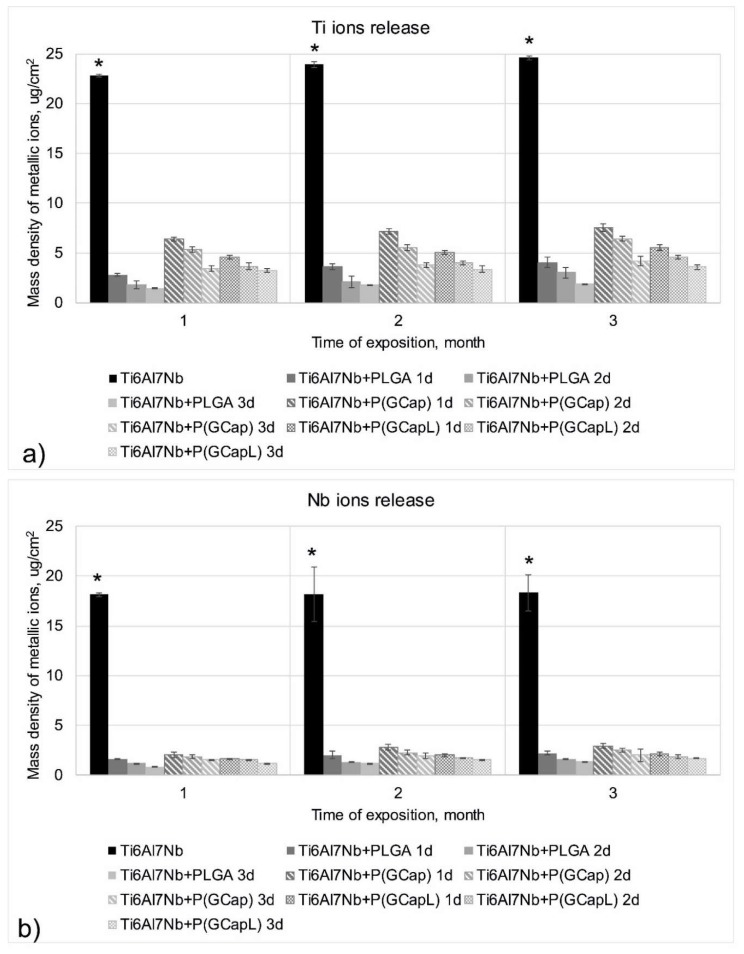
The mean values of the mass of ions density releasing from the surface of the samples coated with a biodegradable polymer coating in the function of the type of the polymer, the parameters of its obtaining, and exposure time: (**a**) Ti ions release and (**b**) Nb ions release (* *p* < 0.05 versus the control group).

The surface density of metal ion mass in Ringer’s solution after one, two, and three months of exposure to Ringer’s solution is shown in Figure 4.

An analysis of the obtained results showed that samples with the Ti6Al7Nb alloy after sandblasting and anodic oxidation without polymer coatings had the highest density of metal ions (Ti, Al, and Nb) releasing to Ringer’s solution. The application of the PLGA, P(GCap), and P(GCapL) polymer coatings caused a significant decrease in the density of ion mass (*p* < 0.05). This proved that the applied polymer coatings constituted a protective barrier that limited the release of the degradation products of the metal substrate into the Ringer’s solution. Preliminary studies of the authors showed a beneficial effect of reducing the mass density of metal ions by biodegradable polymer coatings, which were applied on stainless steel Ti6Al4V and Ti6Al7Nb alloys; however, the applied surface treatment parameters were different than the ones used in the present study [39,47,48]. In the previous work, concerning the PLGA coating (10% w/w) [48], the influence of the number of dips in the process of applying polymer coatings on the mass density of ions releasing into the solution was also observed. In this work, we decided to choose a less-concentrated solution of PLGA-1% to be used for coating preparation in order to verify whether it provid-es the same effect of limiting metal ions. 

Generally, it can be stated that with the increasing number of dips for all analyzed alloying elements of the metal substrate (Ti, Al, and Nb), the surface density of ions decreased significantly (*p* < 0.05). This was visible for Ti and Nb (Figure 4). In the case of aluminum, the application of a polymer coating resulted in a favorable total limitation of the penetration of ions of this element into the solution during the entire measurement period, i.e., after one, two, and three months of exposure, regardless of the number of dips used in the application process. Among the polymer coatings analyzed in the work, the best barrier properties were characterized by the PLGA and P(GCapL) coatings obtained in the dip-coating process consisting of three dips. The reason for this phenomenon may have been the increasing thickness of the produced coating in subsequent dip cycles from one to three. However, an accurate thickness analysis, carried out using the reflectometry method, showed ambiguous results. This was due to the applied pre-treatment process—the sandblasting. Due to the large surface development of the metal substrate obtained during sandblasting, it was difficult to precisely determine the thickness of the coatings by using reflectometry. However, previous unpublished preliminary research has shown that on electrolytically polished 316 LVM (low carbon vacumm melt) steel (whose surface topography led to the estimation of the thickness without any doubts), the number of dips in PLGA solution increases the thickness of the produced coating.

### 3.6. Biological Test Results

Cytotoxicity testing is known to be basic method for establishing the safety of a medical device [49]. This pilot test directs the destiny of the medical device towards further testing, modification, or abandonment at the initial stages of development [50]. Therefore, we conducted a preliminary study to assess the cytotoxicity of the developed coatings. The extract dilution exposure method was used, as it allowed us to detect toxins leached from the exposed surfaces of a wide variety of medical devices [51]. 

**Figure 5:**
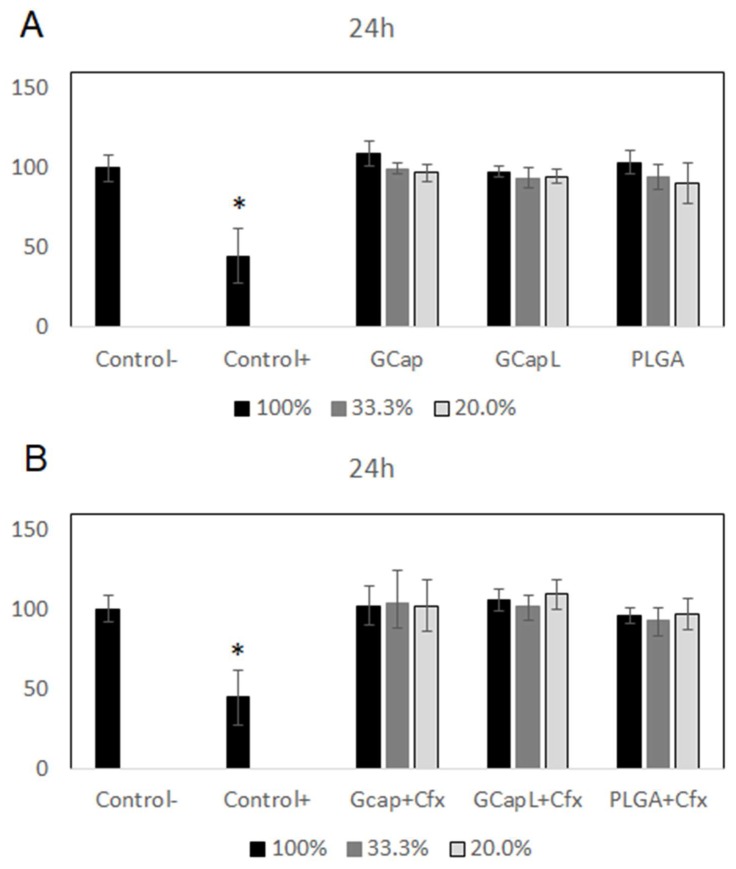
Effect of extracts obtained after 24 h of incubation of the samples with drug-free polymer coatings (**A**) and polymer coatings containing ciprofloxacin (**B**) on the proliferation of cells (* *p* < 0.05 versus the control group).

**Figure 6:**
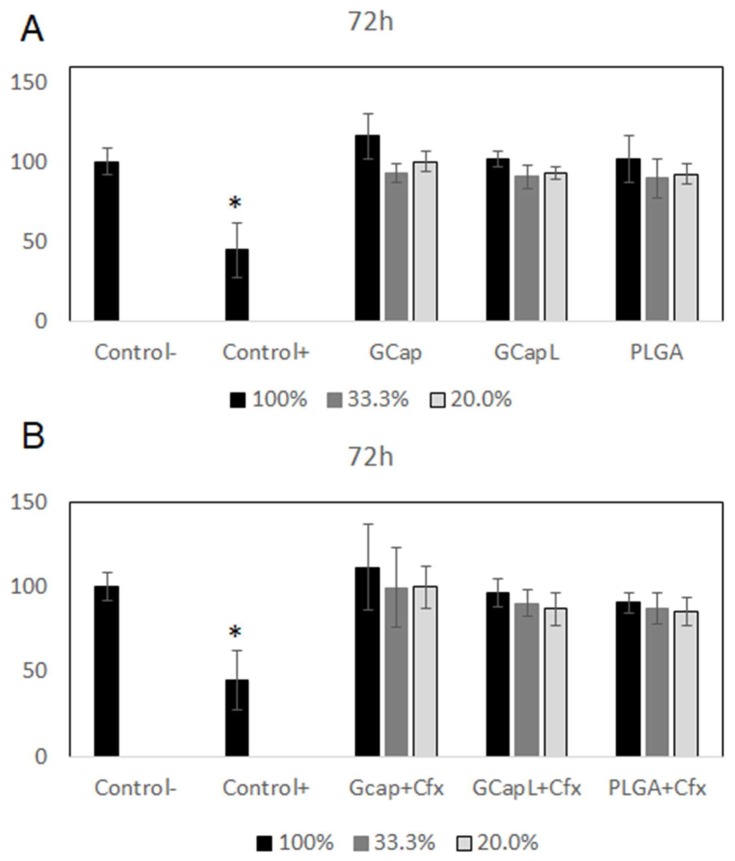
Effect of extracts obtained after 72 h of incubation of the samples with drug-free polymer coatings (**A**) and polymer coatings containing ciprofloxacin (**B**) on the proliferation of cells (* *p* < 0.05 versus the control group).

The extract test revealed that cells cultured with all kinds of polymer extracts revealed similar cell proliferation levels as cells cultured with a blank medium (Figure 5 and Figure 6). The lack of a cytotoxic effect was observed for the whole range of the extract concentrations. As shown in Figure 6, the addition of ciprofloxacin did not affect cell growth compared to untreated cells. The significant decrease of cell proliferation was observed after exposure to a medium with 5% DMSO (positive control).

## 4. Conclusions

The polymers P(GCap), P(GCapL), and PLGA were used to obtain biodegradable coatings deposited on a Ti6Al7Nb alloy. The polymer coatings were enriched with an active substance—ciprofloxacin. The developed coatings were characterized by continuity, homogeneity, translucency, and increased hydrophilicity in relation to the metal substrate. The coatings, regardless of the number of the dips applied, didn’t affected the topography of the substrate, as evidenced by the obtained Sa surface roughness.

As a result of the degradation process of the polymer coatings, which was a consequence of three months of exposure to Ringer’s solution, a gradual change in the physical properties was observed. With a small change in the Sa parameter, the coatings became dull, swell, and their hydrophobicity increased. However, progressive degradation did not reduce the corrosion resistance of the tested Ti alloy. 

In addition, it was found that the application of the polymer coating, regardless of its type, significantly reduced the mass density of ions releasing into the solution. Moreover, it was found that the process of applying polymer layers played an important role in this case. Extract testing was performed to determine the cytotoxic response of cells exposed to the polymer-coated samples. All kinds of drug-loaded and drug-free polymer coatings did not show a decrease in cell proliferation. These results confirmed that there was no cytotoxic response of the cells to the studied materials. 

The comprehensive analysis of the surface properties, along with a detailed characterization of density of the metal ions released during three months of exposure to the corrosive medium, indicated a great potential of modified Ti6Al7Nb alloys with ciprofloxacin-enriched polymer coatings. The developed surface modification intended for short-term implants is economical and easy to obtain, and these should be considered as additional advantages. 

## Figures and Tables

**Table 1:** Surface roughness of the Ti6Al7Nb substrate and Ti6Al7Nb with polymer coatings after different numbers of dips and time of exposition to Ringer’s solution. PLGA: poly (D, L-lactide-glycolide); P(GCap): poly (glycolide-ɛ-caprolactone); and P(GCapL): poly (glycolide-ɛ-caprolactone-L, L-lactide).

The Type of Coating	Surface Roughness Sa, μm				
	Number of Dips (d)	0 months (m) NoE	SD	3 Month (m)	SD
PLGA	1	0.44	0.05	0.49	0.09
	2	0.47	0.03	0.52	0.07
	3	0.45	0.02	0.38	0.07
P(GCap)	1	0.43	0.03	0.48	0.08
	2	0.46	0.04	0.44	0.05
	3	0.39	0.06	0.4	0.06
P(GCapL)	1	0.44	0.02	0.48	0.07
	2	0.39	0.08	0.62	0.06
	3	0.4	0.03	0.39	0.09
Substrate	0	0.47	0.02	0.46	0.03

**Table 2:** Results of potentiodynamic tests.

Sample	Exposition Time, Month	No of Dips	E_corr_, mV	SD	R_p_, MΩ ⋅cm^2^	SD
Ti6Al7Nb	NoE	0	161.9	61	0.84	0.05
	1		108.7	114	0.76	0.02
	2		78.55	48	0.91	0.03
	3		−214.1	5	1.51	0.2
Ti6Al7Nb and PLGA	NoE	1	238.4	240	0.904	0.01
		2	249.35	0.77	0.85	0.03
		3	218.35	53	0.84	0.11
	1	1	132.95	68	0.906	0.09
		2	195.3	6	0.859	0.15
		3	166.2	39	0.98	0.05
	2	1	167.2	3	0.98	0.05
		2	145.45	15	0.88	0.07
		3	153.85	19	0.93	0.01
	3	1	182.3	42	0.875	0.06
		2	238.8	13	0.783	0.04
		3	223.35	38	0.879	0.01
Ti6Al7Nb and P(GCap)	NoE	1	229.45	31	0.92	0.03
		2	48.15	24	0.92	0.03
		3	226.05	3	0.865	0.04
	1	1	−21.5	22	1.04	0.24
		2	83.7	97	1.01	0.37
		3	47.4	34	0.955	0.09
	2	1	85.1	91	1.22	0.04
		2	162.15	55	1.125	0.05
		3	87.95	21	1.09	0.06
	3	1	221	3	0.836	0.01
		2	236.3	5	0.879	0.03
		3	86.65	23	0.789	0.02
Ti6Al7Nb and P(GCapL)	NoE	1	285.3	12	0.868	0.04
		2	275.9	7	0.818	0.01
		3	272	4	0.851	0.01
	1	1	158.9	4	0.969	0.02
		2	83.7	97	1.016	0.37
		3	47.4	34	0.955	0.09
	2	1	169.75	2	0.753	0.01
		2	91.65	38	0.835	0.05
		3	116.6	33	0.766	0.04
	3	1	85.6	78	0.894	0.03
		2	170.75	47	1.017	0.21
		3	174.1	20	0.856	0.04

NoE—no exposition; E_corr_—corrosion potential; R_p_—polarization resistance; and SD—standard deviation.

## References

[1] Dobrzynski P., Li S.M., Kasperczyk J., Bero M., Gasc F., Vert M. (2005). Structure-property relationships of copolymers obtained by ring-opening polymerization of glycolide and epsilon-caprolactone. Part 1. Synthesis and characterization. Biomacromolecules.

[2] Kasperczyk J., Hu Y., Jaworska J., Dobrzyński P., Wei J., Li S. (2008). Comparative study of the hydrolytic degradation of glycolide/l-actide/ε-caprolactone terpolymers initiated by zirconium (IV) acetylacetonate or stannous octoate. J. Appl. Polym. Sci..

[3] Kasperczyk J., Li S., Jaworska J., Dobrzyński P., Vert M. (2008). Degradation of copolymers obtained by ring-opening polymerization of glycolide and ε-caprolactone: A high resolution NMR and ESI-MS study. Polym. Degrad. Stabil..

[4] Maurus P.B., Kaeding C.C. (2004). Bioabsorbable implant material review. Oper. Tech. Sport Med..

[5] Ma X., Xia Y., Xu H., Lei K., Lang M. (2016). Preparation, degradation and in vitro release of ciprofloxacin-eluting ureteral stents for potential antibacterial application. Mater. Sci. Eng. C.

[6] Marciniak J., Szewczenko J., Kajzer W. (2015). Surface modification of implants for bone surgery. Arch. Metall. Mater..

[7] Kajzer A., Kajzer W., Gołombek K., Knol M., Dzielicki J., Walke W. (2016). Corrosion resistance, EIS and wettability of the implants made of 316 LVM steel used in chest deformation treatment. Arch. Metall. Mater..

[8] Krauze A., Ziębowicz A., Marciniak J. (2005). Corrosion resistance of intramedullary nails used in elastic osteosynthesis of children. J. Mater. Process. Tech..

[9] Makuch K., Koczorowski R. (2010). Biokompatybilność tytanu oraz jego stopów wykorzystywanych w stomatologii. Dent. Med. Probl..

[10] Rusinek B., Stobiecka A., Obtułowicz K. (2008). Alergia na tytan i implant. Alergol. Immunologia..

[11] Zhang Y., Xiu P., Jia Z., Zhang T., Yin C., Cheng Y., Cai H., Zhang K., Song C., Leng H. (2018). Effect of vanadium released from micro-arc oxidized porous Ti6Al4V on biocompatibility in orthopedic applications. Colloids Surf. B.

[12] Ye S.-H., Johnson C.A., Wooley J.R., Murata H., Gamble L.J., Ishihara K., Wagner W.R. (2010). Simple surface modification of a titanium alloy with silanated zwitterionic phosphorylcholine or sulfobetaine modifiers to reduce thrombogenicity. Colloids Surf. B.

[13] Roessler S., Zimmermann R., Scharnweber D., Werner C., Worch H. (2002). Characterization of oxide layers on Ti6Al4V and titanium by streaming potential and streaming current measurements. Colloids Surf. B.

[14] Basiaga M., Paszenda Z., Walke W., Karasiński P., Marciniak J. (2014). Electrochemical Impedance Spectroscopy and corrosion resistance of SiO2 coated cpTi and Ti-6Al-7Nb alloy. Information Technologies in Biomedicine, Advances in intelligent Systems and Computing.

[15] Basiaga M., Walke W., Paszenda Z., Kajzer A. (2016). The effect of EO and steam sterilization on the mechanical and electrochemical properties of titanium Grade 4. Mater. Technol..

[16] Kiel-Jamrozik M., Szewczenko J., Basiaga M., Nowińska K. (2015). Technological capabilities of surface layers formation on implant made of Ti-6Al-4V ELI alloy. Acta Bioeng. Biomech..

[17] Szewczenko J., Nowinska K., Marciniak J. (2011). Influence of initial surface treatment on corrosion resistance of Ti6Al4V ELI alloy after anodizing. Prz. Elektrotechniczn..

[18] Park M., Lee J., Park Ch Lee S., Seok H., Choy Y. (2013). Polycaprolactone coating with varying thyicknesses for controlled corrosion of magnesium. J. Coat. Technol. Res..

[19] Xu W., Yagoshi K., Koga Y., Sasaki M., Niidome T. (2018). Optimized polymer coating for magnesium alloy-based bioresorbable scaffolds for long-lasting drug release and corrosion resistance. Colloids Surf. B.

[20] Wu W., Zhou Z., Liu W., Zhao Y., Zhao Y., Huang T., Li X., Fang J. (2019). Preparation and In-vitro Degradation Behavior of Poly(L-lactide-co-glycolide-co-ε-caprolactone) Terpolymer. J. Macromol. Sci. B.

[21] Ulery B.D., Nair L.S., Laurencin C.T. (2011). Biomedical applications of biodegradable polymers. J. Polym. Sci. B Polym. Phys..

[22] Dash T.K., Konkimalla V.B. (2012). Poly-ϵ-caprolactone based formulations for drug delivery and tissue engineering: A review. J. Control. Release.

[23] Müller A., Ávila M., Saenz G., Salazar J., Jiménez A., Peltzer M., Ruseckaite R. (2015). Crystallization of PLA-based Materials. Poly(Lactic Acid) Science and Technology: Processing, Properties, Additives and Applications.

[24] Liu H., Wang S.D., Qi N. (2012). Controllable structure, poperties and degradation of the electrospun PLGA/PLA-Blended nanofibrous scaffolds. J. Appl. Polym. Sci..

[25] Jelonek K., Kasperczyk J. (2013). Polyesters and polyestercarbonates for controlled drug delivery. Polimery.

[26] Cieślik M., Engvall K., Pan J., Kotarba A. (2011). Silane-parylene coating for improving corrosion resistance of stainless steel 316L implant material. Corros. Sci..

[27] Cieślik M., Kot M., Reczyński W., Engvall K., Rakowski W., Kotarba A. (2012). Parylane coatings on stainless steel 316L Surface for medical applications—Mechanical and prottective properties. Mater. Sci. Eng. C.

[28] Cieślik M., Zimowski S., Gołda M., Engvall K., Pan J., Rakowski W., Kotarba A. (2012). Engineering of bone fixation metal implants biointerface—Application of parylene C as versatile protective coating. Mater. Sci. Eng. C.

[29] Kazek-Kęsik A., Jaworska J., Krok-Borkowicz M., Gołda-Cępa M., Pastusiak M., Brzychczy-Włoch M., Pamuła E., Kotarba A., Simka W. (2016). Hybrid oxide-polymer formed on Ti-Mo alloy surface enhancing antibacterial and osseointegration functions. Surf. Coat. Tech..

[30] Orchel A., Jelonek K., Kasperczyk J., Dobrzyński P., Marcinkowski A., Pamuła E., Orchel J., Bielecki I., Kulczycka A. (2013). The influence of chai microstructure of biodegradable copolyesters obtained with low-toxic zirconium initiator to in vitro biocompatibility. BioMed Res. Int..

[31] Vogeling H., Duse L., Seitz B.S., Plenagl N., Wojcik M., Pinnapireddy S.R., Bakowsky U. (2018). Multilayer bacteriostatic coating for surface modified titanium implant. Phys. Status Solidi A.

[32] Zhang G.-F., Liu X., Zhang S., Pan B., Liu M.-L. (2018). Ciprofloxacin derivatives and their antibacterial activities. Eur. J. Med. Chem..

[33] Jaworska J., Jelonek K., Jaworska-Kik M., Musiał-Kulik M., Marcinkowski A., Szewczenko J., Kajzer W., Pastusiak M., Kasperczyk J. (2020). Development of antibacterial, ciprofloxacin-eluting biodegradable coatings on Ti6Al7Nb implants to prevent peri-implant infections. J. Biomed. Mater. Res. Part A.

[34] Standard ISO 5832-11:2014 (2014). Implants for Surgery—Metallic Materials—Part 11: Wrought Titanium 6-Aluminium 7-Niobium Alloy.

[35] Zini E., Scandola M., Dobrzynski P., Kasperczyk J., Bero M. (2007). Shape memory behavior of novel (L-Lactide- glycol ide-trimethylene carbonate) terpolymers. Biomacromolecules.

[36] Standard PN EN ISO 10993-15:2009 (2009). Biological Evaluation Of Medical Devices—Part 15: Identification And Quantification Of Degradation Products From Metals And Alloys.

[37] Standard ISO 10993-5:2009 (2009). Biological Evaluation of Medical Devices—Part 5: Tests for in Vitro Cytotoxicity.

[38] Kazek-Kęsik A., Nosol A., Płonka J., Śmiga-Matuszowicz M., Gołda-Cępa M., Krok-Borkowicz M., Brzychczy-Włoch M., Pamuła E., Simka W. (2019). PLGA-amoxicillin-loaded layer formed on anodized Ti alloy as a hybrid material for dental implant applications. Mater. Sci. Eng. C.

[39] Szewczenko J., Kajzer W., Grygiel-Pradelok M., Jaworska J., Jelonek K., Gawliczek M., Libera M., Marcinkowski A., Kasperczyk J. (2017). Corrosion resistance of PLGA-coated biomaterials. Acta Bioeng. Biomech..

[40] Li S., Vert G.M. (1990). Structure-property relationship in the case of the degradation of massive poly(α-hydroxyacids) in aqueous media – Part 2 Degradation of lactide-glycolide copolymers PLA37.5GA25 and PLA75GA25. J. Mater. Sci.-Mater. Med..

[41] Li S., Vert G.M. (1990). Structure-property relationship in the case of the degradation of massive poly(α-hydroxyacids) in aqueous media – Part 1: Poly(dl-lactic acid). J. Mater. Sci.-Mater. Med..

[42] Saad B., Suter U.W., Buschow K.H.J., Cahn R.W., Flemings M.C., Ilschner B., Kramer E.J., Mahajan S., Veyssière P. (2001). Biodegradable polymeric materials. Encyclopedia of Materials: Science and Technology.

[43] Huang H.-H., Wu C.-P., Sun Y.-S., Lee T.-H. (2013). Improvements in the corrosion resistance on biocompatibility of biomedical Ti6Al7Nb alloy using an electrochemical anodization treatment. Thin Solid Films.

[44] Stoica E.-D., Fedorov F., Nicolae M., Uhlemann M., Gebert A., Shultz L. (2012). Ti6Al7Nb surface modification by anodization in electrolytes containing HF. Sci. Bull.-Univ.Politeh. Buchar. Ser. B.

[45] de Assi S.L., Wolynec S., Costa I. (2006). Corrosion characterization of titanium alloys by electrochemical techniques. Electrochim. Acta.

[46] Lavos-Valereto I.C., Wolynec S., Ramires I., Guastaldi A.C., Costa I. (2004). Electrochemica impedance spectroscopy, characterization of passive film formed on implant Ti6Al7Nb alloy in Hank’s solution. J. Mater. Sci.-Mater. Med..

[47] Kajzer W., Jaworska J., Jelonek K., Szewczenko J., Kajzer A., Nowińska K., Hercog A., Kaczmarek M., Kasperczyk J. (2018). Corrosion resistance of Ti6Al4V alloy coated with caprolactone-based biodegradable polymeric coatings. Eksploat. Niezawodn..

[48] Szewczenko J., Kajzer W., Kajzer A., Basiaga M., Kaczmarek M., Major R., Simka W., Jaworska J., Jelonek K., Karpeta-Jarząbek P. (2019). Adhesion of poly(lactide-glycolide) coating (PLGA) on the Ti6Al7Nb alloy Substrate. Proceedings of the International Conference on Information Technolgy in Biomedicine.

[49] Li W.J., Zhou J., Xu Y.Y. (2015). Study of the in vitro cytotoxicity testing of medical devices. Biomed. Rep..

[50] Fricker S.P. (1994). A role for in-vitro cytotoxicity testing in the selection and development of metal-based pharmaceutical and materials products. Toxicol. in Vitro.

[51] Srivastava G.K., Alonso-Alonso M.L., Fernandez-Bueno I., Garcia-Gutierrez M.T., Rull F., Medina J., Coco R.M., Pastor J.C. (2018). Comparison between direct contact and extract exposure methods for PFO cytotoxicity evaluation. Sci. Rep.-U. K..

